# Predicting the amputation risk for patients with diabetic foot ulceration – a Bayesian decision support tool

**DOI:** 10.1186/s12911-020-01195-x

**Published:** 2020-08-24

**Authors:** Jens Hüsers, Guido Hafer, Jan Heggemann, Stefan Wiemeyer, Swen Malte John, Ursula Hübner

**Affiliations:** 1 Health Informatics Research Group, Department of Business Management and Social Sciences, University of Applied Sciences Osnabrück, Osnabrück, Germany; 2 Niels Stensen Kliniken, Christliches Klinikum, Melle, Germany; 3 Department Dermatology, Environmental Medicine, Health Theory, University of Osnabrück, Osnabruck, Germany

## Abstract

**Background:**

Diabetes mellitus is a major global health issue with a growing prevalence. In this context, the number of diabetic complications is also on the rise, such as diabetic foot ulcers (DFU), which are closely linked to the risk of lower extremity amputation (LEA). Statistical prediction tools may support clinicians to initiate early tertiary LEA prevention for DFU patients. Thus, we designed Bayesian prediction models, as they produce transparent decision rules, quantify uncertainty intuitively and acknowledge prior available scientific knowledge.

**Method:**

A logistic regression using observational collected according to the standardised PEDIS classification was utilised to compute the six-month amputation risk of DFU patients for two types of LEA: 1.) any-amputation and 2.) major-amputation. Being able to incorporate information which is available before the analysis, the Bayesian models were fitted following a twofold strategy. First, the designed prediction models waive the available information and, second, we incorporated the a priori available scientific knowledge into our models. Then, we evaluated each model with respect to the effect of the predictors and validity of the models. Next, we compared the performance of both models with respect to the incorporation of prior knowledge.

**Results:**

This study included 237 patients. The mean age was 65.9 (SD 12.3), and 83.5% were male. Concerning the outcome, 31.6% underwent any- and 12.2% underwent a major-amputation procedure. The risk factors of perfusion, ulcer extent and depth revealed an impact on the outcomes, whereas the infection status and sensation did not. The major-amputation model using prior information outperformed the uninformed counterpart (AUC 0.765 vs AUC 0.790, Cohen’s d 2.21). In contrast, the models predicting any-amputation performed similarly (0.793 vs 0.790, Cohen’s d 0.22).

**Conclusions:**

Both of the Bayesian amputation risk models showed acceptable prognostic values, and the major-amputation model benefitted from incorporating a priori information from a previous study. Thus, PEDIS serves as a valid foundation for a clinical decision support tool for the prediction of the amputation risk in DFU patients. Furthermore, we demonstrated the use of the available prior scientific information within a Bayesian framework to establish chains of knowledge.

## Background

Diabetes mellitus is a major global health issue [1, 2]. Reflected by the high prevalence rates in the past [3–5], diabetes presently is and presumably will remain a significant challenge for societies and health systems worldwide, as studies forecast increasing prevalence rates for the upcoming years [6–8].

Along with high prevalence rates, the number of diabetic complications also increases [9], among them are diabetic foot ulceration (DFU) [10]. Closely linked to the risk of lower extremity amputation, DFU causes imminent disease burden, high costs [11, 12] and high mortality rates [13–15]. While, in medical care and nursing, the prevention of DFU should be the primary imperative [16], it is essential to identify DFU patients with a high amputation risk early in order to initiate close ulcer monitoring and care, including amputation-preventive actions. In this context, ensuring comparable clinical statements about ulcers, standardised classification systems are used for cross-sectional and longitudinal ulcer documentation. Those classifications are widely used in routine clinical care and research. Existing studies that have investigated the prognostic value of these classifications in DFU care promised to provide a foundation for decision support systems used for amputation risk modelling [17, 18]. For example, as part of the multi-centre EURODIALE initiative, Pickwell et al. developed a well-performing risk assessment tool for amputation based on the PEDIS classification [18]. PEDIS reliably describes [19–21] diabetic foot according to five categories, i.e. risk factors, which form the respective acronym: **p**erfusion status, **e**xtent of ulcer, **d**epth of ulcer, **i**nfection status and **s**ensation [19, 22] (see Table 1). In the approach of Pickwell et al., each category is assessed, scored and then aggregated to a final sum so that a larger overall score correlates with higher amputation risk.

**Table 1:** We used the PEDIS classification system developed by the International Working Group on the Diabetic Foot (IWGDF).The table gives an overview of the PEDIS classification. For more detail, please refer to Schaper (2004) [23]. Additional information about the PEDIS assessment is given in the method section of this study

Grade/ Score	Perfusion	Extent	Depth	Infection	Sensation
1	No peripheral arterial disease (PAD)	< 1 cm^2^	Superficial	No symptoms or sign of infection	No loss of protective sensation
2	PAD, No critical limb ischaemia (CLI)	1–5 cm^2^	Fascia, muscle, tendon	Infection involving the skin and the subcutaneous tissue only	Loss of protective sensation
3	CLI	> 5 cm^2^	Bone or joint	Erythema > 2 cm plus one of: swelling, tenderness, warmth, discharge; or infection involving structures deeper than skin and subcutaneous tissues	
4				Systemic inflammatory response syndrome (SIRS)	

In contrast, prognostic systems developed with statistical modelling techniques may achieve higher validity compared to scoring systems. Furthermore, and opposed to sum scores, these statistical models can compute a probability value expressing the amputation risk, whose interpretation might be more convenient for clinicians [24].

In this light, we aimed to design prediction models based on the PEDIS classification using a logistic regression model, which computes the probability value for the amputation. For model development, a Bayesian modelling approach lends itself because it offers fundamental advantages that are important in the domain of clinical predictive modelling. First, Bayesian models are transparent as they provide model details represented by complete probability distributions about estimated model parameters, statistical metrics, such as AUC values, and predictions [25, 26]. This yields transparency, allowing clinical users of a model to understand the decision rules and better grasp the predicted results in order to embody them into their clinical reasoning and clinical action safely [27, 28].

Second, Bayesian models can incorporate a priori knowledge, i.e. information about the model parameters available before the analysis. This fundamental feature enables Bayesian analysis to serve as a framework for a learning health system characterised by a collaborative, interorganisational, data-driven research process [29, 30]. In this way, without sharing sensitive patient data, researchers can directly incorporate prior available scientific information into their statistical models, thereby accumulating knowledge by statistically combining existing research and their data. Still, this requires similar research questions and standardised structured data across research initiatives. As the PEDIS classification is a standardised and widely accepted classification system, it serves as a useful showcase in developing and validating Bayesian predictive models for clinical decision making.

Thus, this study follows three research questions: First, what is the effect of each of the PEDIS risk factors on the outcome, i.e. amputation? Second, what is the prognostic value of PEDIS prediction models? Furthermore, we intend to investigate the impact of prior knowledge. Therefore, we plan to design and compare models that waive prior knowledge with models that incorporate available knowledge. Accordingly, the third research question is: Does the incorporation of prior available scientific knowledge improve the prognostic value of the models?

## Methods

### Study design and sample

We conducted an observational, prospective single-centre, open cohort study, including Diabetes Mellitus (DM) patients with a diabetic foot ulcer (DFU).

The study pursued a prospective study design, as the data collection and analysis were planned before the eligible subjects were studied. The investigation was conducted at a specialised wound care centre as part of the traumatic surgery unit of Klinikum Melle in Germany, which is a partner in a local learning health system. Data capture started on 1st June 2013 and ended on 1st July 2019. We recruited male and female type 1 and 2 diabetes patients who were over 18 years old. The exclusion criteria were traumatic wounds, tumour-induced ulcers and patients without DM. GH screened all inpatient and outpatient subjects with DFU before study entry.

At baseline, GH, JHe, and SW assessed all the included patients according to the PEDIS classification (IWGDF) as described by Schaper [23]. To obtain the perfusion status, for which different methods are described [23], e.g. assessing the tcoO2 value, we used the blood pressure as an indicator, i.e., the Ankle Brachial Pressure Index (ABPI) and systolic ankle pressure. To obtain the sensation, we used 10 g-monofilament (on three sites of the plantar foot) and a 128 Hz tuning fork on the hallux. When a patient had more than one ulcer, we chose the one with the highest PEDIS score as the index ulcer. Gender and age at baseline were collected s demographic variables. After the baseline assessment, patients were treated in the wound care centre according to the national clinical guidelines [31] by an interdisciplinary team of physicists (surgeons, diabetologists, cardiologists), specialised nurses, physiotherapists, podiatrists. To ensure an optimal healing situation, an external orthopaedic technician supplied patients with individual offloading systems on-site in close collaboration with the wound care centre. Six months after each patient’s baseline assessment, we determined the final treatment outcomes as any-amputation (ICD-9 CM code: 84.13–84.19 and 84.11–84.12) and major-amputation (amputations above the ankle ICD-9 CM code 84.11–84.12), however, as in accordance with Pickwell et al. [18], excluding the lesser toes.

### Statistical analysis

To answer the first two research questions, Bayesian models were fitted using the five PEDIS risk factors as the predictors as well as age and gender as the covariates. Each of the clinical outcomes served as the criterion. With regard to the third research question, focusing on the effect of prior knowledge, we followed a twofold strategy. First, we created models for both outcomes waiving prior knowledge. Second, we fitted the same models, but in this step, we incorporated the available information.

We used prior information for three main reasons: First, prior knowledge helps to make more reliable inferences when only a small amount of data is available. Second, as prior knowledge mainly represents external scientific knowledge, it is especially useful for our risk models as we utilised data from a single wound care centre. Third, as the role of the PEDIS risk factors is established in clinical care and research, a models waiving prior information neglect existing knowledge and would stand against the Bayesian reasoning, encouraging the use of the available knowledge in these situations [25, 32].

**Fig. 1:**
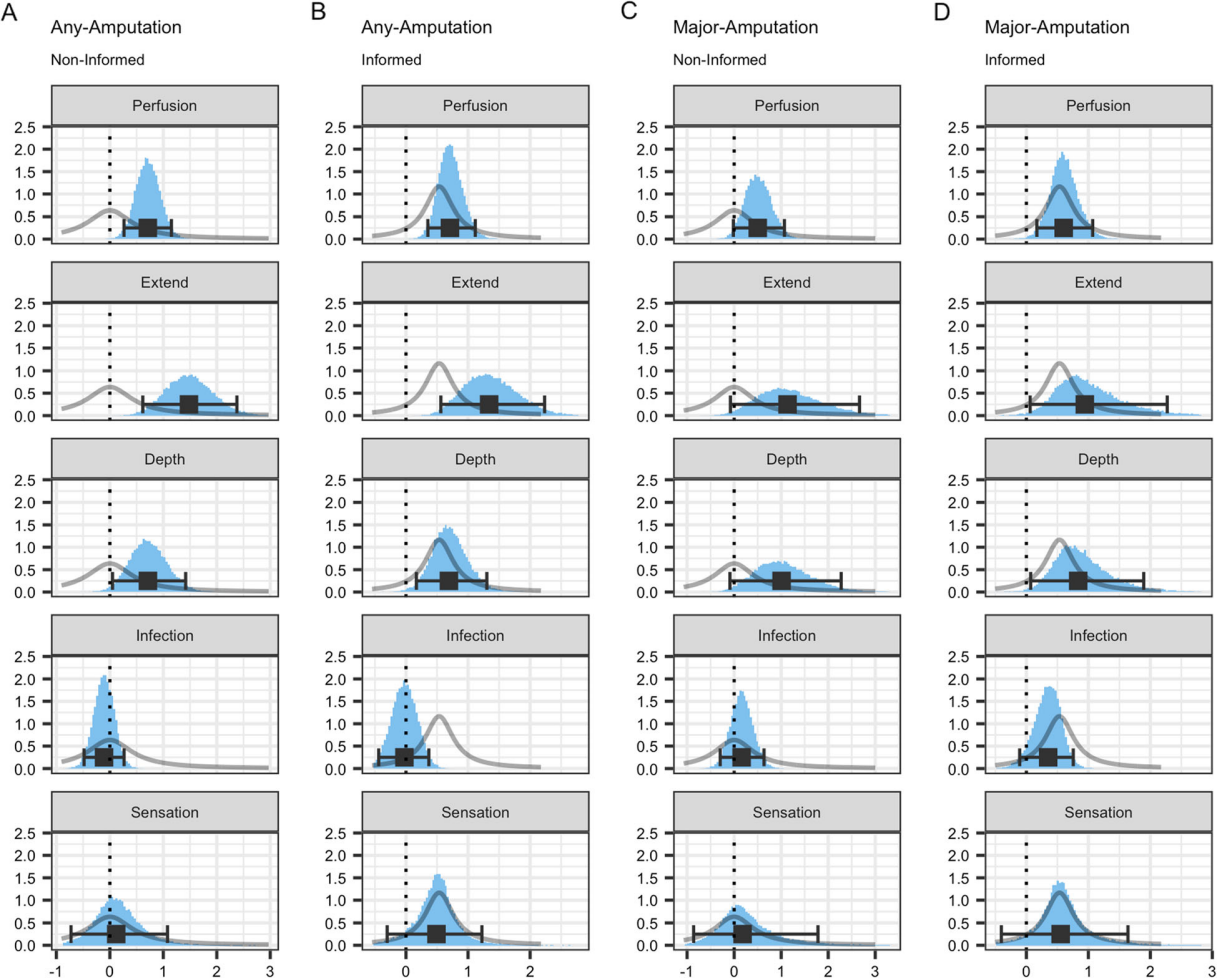
A histogram matrix of the posterior coefficient distributions of all four model. Each column shows a model; each row shows a risk factor. The light blue histograms show the posterior distributions. The light grey density curves represent the prior probability distribution of the models. The black error bar shows the 95% highest density interval (HDI). The black square in the middle of the error bar represents the posterior median estimate of the model coefficients. The dotted vertical line represents the null-value of 0, which, when exponentiated, corresponds to an OR of 1). Risk models: column **a** uninformed any-amputation; column **b** informed any-amputation; column **c** uninformed major-amputation; column **d** informed major-amputation

The priors were designed with Cauchy distributions as proposed by Gelman et al. [33]. In the first approach, i.e. modelling without prior information, we assigned a zero-centred Cauchy distribution with a broad scale of 1 to each predictor, spanning a vast range of prior plausible model coefficients. In the second case, i.e. with knowledge, odds ratios and corresponding confidence intervals (CI) were derived from the published data in the study of Pickwell et al. [18] which were then pooled and further adjusted by subtracting the standard error twice. Thereby, the odds ratios were designed as conservative prior estimates reflecting the general pattern of external knowledge. The log-transformed odds ratios were then integrated as parameters into informative Cauchy distributions for the Bayesian logistic regression. Figure 1 illustrates the implemented prior distributions.

The Monte Carlo Markov Chain (MCMC) sampling procedure was used to fit the Bayesian models, and for the computation of the posterior distributions, the open-source R-package *rstanarm* (version 2.19.2) was used [34]. For each model, we generated four MCMC chains with 8500 steps and set the burn-in period to 500 iterations, yielding 30,000 sample MCMC steps for each model.

To make inferences about the effect of the risk factors on the outcome, 95% Highest Density Intervals (HDI) were computed as credible intervals, which are common in Bayesian statistics [25, 35]. Any value within the interval has a higher density than the values outside, and the total mass of values inside is 95%. As the HDI contains the 95% most credible posterior values of the predictor, it is considered to be associated with the outcome when the HDI excludes the null value [35, 36]. For beta-coefficients, the null value is zero, but it is also common to exponentiate beta coefficients in logistic regression, whereby they can be interpreted as odds ratios [37]. In this case, the null value is one.

We used the programming language R (version 3.6.2) and additional open-source R-packages for all the statistical analyses [34, 38–40].

## Results

### Sample description

A total of 254 patients met the inclusion criteria and were recorded at baseline (admission to the wound care centre and inpatients). Sixteen patients were lost to follow-up (deceased or dropped out due to other reasons, e.g. moved), which resulted in 237 study participants.

The mean age of the sample was 65.9 (SD 12.3) years. The proportion of female and male patients was 16.5 and 83.5%, respectively. Among all the patients, 31.6% (*n* = 75) had at least any-amputation (excluding lesser toes) and 12.2% (*n* = 29) underwent a major-amputation procedure (Table 2).

**Table 2:** Descriptive summary of the age, gender, the PEDIS sum score and each of the PEDIS risk factors for the overall sample, any-amputation and major-amputation status. Data are shown in per cent; except the PEDIS sum score and age, which are summarised by the mean. The final count of any-amputees is the sum of the minor- (*n* = 46) and major amputations (*n* = 29)

Characteristic	Grade/ Score	Overall Sample		Any-Amputation				Major-Amputation			
				Non Amputees (***n*** = 162, 68.4%)		Amputees* (***n*** = 75, 31.6%)		Non Amputees (***n*** = 208, 87.8%)		Amputees (***n*** = 29, 12.2%)	
		Proportion	Frequency (***n*** = 237)	Proportion	n	Proportion	n	Proportion	n	Proportion/ Mean	n
**Gender**	**Female**	16.46%	39	12.24%	29	4.22%	10	14.77%	35	1.69%	4
	**Male**	83.54%	198	56.12%	133	27.43%	65	73%	173	10.55%	25
**Age, years (mean)**	**–**	65.91 (SD 12.3)	237	64.59 (SD 12.8)	162	68.76 (SD 10.5)	75	65.55 (SD 12.5)	208	68.52 (SD 12.4)	29
**PEDIS Sum (mean)**	**–**	10.76 (SD 1.9)	237	10.27 (SD 1.9)	162	11.81 (SD 1.4)	75	10.56 (SD 1.9)	208	12.17(SD 1.4)	29
**Perfusion**	**Stage 1**	48.1%	114	40.93%	97	7.17%	17	45.57%	108	2.53%	6
	**Stage 2**	32.49%	77	20.25%	48	12.24%	29	27.85%	66	4.64%	11
	**Stage 3**	19.41%	46	7.17%	17	12.24%	29	14.35%	34	5.06%	12
**Extent**	**Stage 1**	3.38%	8	3.38%	8	0%	0	3.38%	8	0%	0
	**Stage 2**	31.22%	74	28.27%	67	2.95%	7	30.38%	72	0.84%	2
	**Stage 3**	65.4%	155	36.71%	87	28.69%	68	54.01%	128	11.39%	27
**Depth**	**Stage 1**	5.91%	14	5.06%	12	0.84%	2	5.91%	14	0%	0
	**Stage 2**	32.91%	78	28.27%	67	4.64%	11	31.65%	75	1.27%	3
	**Stage 3**	61.18%	145	35.02%	83	26.16%	62	50.21%	119	10.97%	26
**Infection**	**Stage 1**	36.29%	86	26.16%	62	10.13%	24	33.76%	80	2.53%	6
	**Stage 2**	35.44%	84	22.78%	54	12.66%	30	29.54%	70	5.91%	14
	**Stage 3**	25.74%	61	18.14%	43	7.59%	18	22.78%	54	2.95%	7
	**Stage 4**	2.53%	6	1.27%	3	1.27%	3	1.69%	4	0.84%	2
**Sensation**	**Stage 1**	7.17%	17	5.49%	13	1.69%	4	6.75%	16	0.42%	1
	**Stage 2**	92.83%	220	62.87%	149	29.96%	71	81.01%	192	11.81%	28

### Model diagnostics

We reviewed the Markov Chain Monte Carlo (MCMC) sampling procedure of the Bayesian logistic regression models for convergence, autocorrelation, effective sample size (ESS) and collinearity. The chain of each coefficient converged, lacked autocorrelation and showed a large effective sample size (> 10,000) for all the model coefficients (see Additional file 1). The covariate matrix revealed an absence of collinearity (see Additional file 2). Univariate models, which were calculated to explore the relationships, showed that perfusion, extent and depth had an impact on the outcome while infection and sensation were unconnected with the outcome (see Additional file 3). Based on the MCMC sample, we were able to compute the posterior coefficient distributions, posterior AUC distributions as well as distribution of the predictions (Script available in Additional file 4).

### Models without prior knowledge

The any-amputation risk model provided significant evidence of a positive effect of perfusion status, ulcer extent and ulcer depth as their 95% HDI excluded the null value (Table 3). In contrast, the 95% HDI of infection status and loss of sensation covered the null value and thus indicated no impact.

**Table 3:** Summary of the four PEDIS risk models. To support a more straightforward interpretation of the **logistic regression model coefficients**, we present them as **odds ratios (OR)** in this table; the logistic regression coefficients can be interpreted as odds ratios when exponentiated. The OR is the exponentiated median of the posterior coefficient distribution. Furthermore, **the 95% HDI of the OR** is presented. HDI is derived from the posterior distribution. Any value within the interval has a higher density than the values outside the HDI. The total mass of values inside the HDI is 95%

Risk Factors (Predictors)	Any Amputation				Major Amputation			
	Non-Informed Model		Informed Model		Non-Informed Model		Informed Model	
	Beta-Coefficients	Odds Ratios	Beta-Coefficients	Odds Ratios	Beta-Coefficients	Odds Ratios	Beta-Coefficients	Odds Ratios
Perfusion	0.688 [0.264–1.152]	1.990 [1.302–3.164]	0.703 [0.352–1.116]	2.020 [1.422–3.052]	0.471 [−0.017–1.069]	1.601 [0.983–2.913]	0.586 [0.170–1.070]	1.798 [1.185–2.914]
Extend	1.484 [0.617–2.376]	4.411 [1.853–10.762]	1.283 [0.562–2.233]	3.609 [1.754–9.326]	0.985 [−0.079–2.665]	2.678 0.924–14.364]	0.799 [0.061–2.272]	2.222 [1.063–9.702]
Depth	0.665 [0.050–1.418]	1.945 [1.051–4.128]	0.656 [0.168–1.302]	1.927 [1.183–3.677]	0.932 [− 0.088–2.275]	2.540 [0.916–9.726]	0.694 [0.069–1.893]	2.001 [1.071–6.642]
Infection	− 0.112 [− 0.483–0.266]	0.894 [0.617–1.305]	−0.021 [− 0.439–0.368]	0.979 [0.644–1.446]	0.155 [− 0.296–0.635]	1.167 [0.744–1.888]	0.368 [− 0.108–0.757]	1.445 [0.898–2.131]
Sensation	0.037 [− 0.726–1.077]	1.037 [0.484–2.937]	0.516 [− 0.303–1.223]	1.675 [0.738–3.397]	0.076 [−0.861–1.782]	1.079 [0.423–5.943]	0.553 [− 0.404–1.639]	1.738 [0.668–5.149]
AUC Value	0.793		0.790		0.765		0.790	
AUC HDI	[0.778–0.801]		[0.774–0.802]		[0.725–0.779]		[0.774–0.802]	

The major-amputation risk model lacked significant evidence of an effect of any PEDIS risk factor as their 95% HDIs included the null value. However, the proportion of posterior coefficient values below zero for perfusion, extent and depth was 2.7, 2 and 2.2%, respectively, which hints at a positive relationship as most posterior values cluster above zero (Fig. 1 and Table 3).

Concerning the prognostic value, the receiver operating characteristic analysis of the posterior AUC distributions showed a posterior median estimate of 0.793 (95% HDI 0.778–0.801) for any-amputation and 0.765 (95% HDI 0.725–0.779) for major-amputation. Comparing both AUC values (Δ = 0.031), the former model revealed a higher predictive value.

### Models including prior knowledge

To evaluate the impact of informative priors, we compared the Bayesian models, which used a neutral prior and were presented in the last sections, with models which incorporated external, a priori available scientific knowledge.

The informative priors had an impact on the major-amputation risk model, which mainly led to a decreased variance in the posterior coefficient distribution (Table 3). As a result, the informed model revealed three non-null predictors: perfusion status, ulcer extent and depth. The decreased variance also affected the predictive value of the informed model for the outcome major-amputation: Yielding a large effect size (Cohen’s d = 2.217), the difference between both AUC values (Δ = 0.029) suggested that the informed model outperformed the uninformed counterpart (AUC: 0.765 vs 0.790).

Opposed to the major-amputation models, the bottom-line finding for the any-amputation models remained the same: the 95% HDI of perfusion, extent and depth excluded the neutral value of zero (which corresponds to the neutral odds ratio of 1 when exponentiated), whereas those for infection and sensation did not (Table 3 and Fig. 1). The posterior median estimates for the coefficients and the AUC remained nearly constant, e.g. AUC: 0.793 vs 0.790 (Cohen’s d = 0.224, Δ = 0.003).

### Model application for decision support

The results of these models can be visualised for clinical use to support decision making. Figure 3 shows the distributions of the predicted probabilities for any-amputation using the informed model for two hypothetical ulcers classified according to the PEDIS system. In the first case, the PEDIS classification describes a rather moderate ulcer. In this first example, the patient has a peripheral arterial disease but no critical limb ischaemia (stage 2/3), an ulcer extent ranging between one and five cm^2^ (stage 2/3), but just a superficial ulcer (stage 1/3), no signs of infection (stage 1/4) and the presence of protective sensation (stage 1/2). In this case, the model computes a median posterior amputation risk of 4.4% (95% HDI 0.2–13%). The second example describes a more severe ulcer, i.e. the limb perfusion is critical due to ischaemia (stage 3/3), the ulcer extends > five cm^2^ (stage 3/3) and it reaches deep tissues (stage 3/3), and infection (stage 1/4) and sensation status (stage 1/2) (Table 1). In this case, the median posterior risk estimate was 63% (95% HDI 37.2–85.9%).

The model clearly discriminates between both diabetic ulcers predicting a lower risk for the former compared to the latter which is not only indicated by the median risk prediction but also in the non-overlapping HDIs. Furthermore, the posterior variability quantified by the HDI expresses the associated uncertainty of the predicted risk: The range of the 95% HDI of the former example is nearly four times narrower than that of the latter and, therefore, the model is more confident in its former prediction.

## Discussion

### Summary

In the present study, we developed amputation risk models based on the PEDIS classification system using Bayesian statistics. Bayesian approaches are becoming more popular in applied research [41] and they open an entirely new avenue for the interpretation of model parameters and enable the establishment of chains of knowledge by incorporating prior available scientific information.

Including 237 patients with DFU, we examined the effect of each PEDIS risk factor and the outcome variables any- and major-amputation. Furthermore, we conducted a receiver operating characteristic analysis to assess the prognostic value of the models. In summary, except for the non-informed major-amputation model, the posterior coefficient distributions of the other models revealed a positive effect of the three predictors: perfusion status, ulcer extent and ulcer depth. Among both outcomes, the PEDIS risk model for any-amputation had a slightly higher prognostic value compared to the risk model for major-amputation.

In addition, we investigated the impact of prior available scientific knowledge on the association of PEDIS risk factors with the outcomes as well as on the predictive value of the models. The major-amputation risk model could be improved through the inclusion of prior information, which manifested in non-null coefficients (perfusion status, ulcer extent and ulcer depth) and improved the prognostic value. In contrast, prior incorporated knowledge had no impact on the any-amputation risk model.

Our risk models did not provide enough evidence of an impact of the two remaining PEDIS risk factors infection status and sensation. This stands in contrast with previous studies [42] but confirms our univariate findings. However, as the risk factor infection is a red flag, it may have facilitated close ulcer monitoring and attentive care as an amputation protective measure. Furthermore, this finding may be the result of the categorical design of the PEDIS risk factors and the loss of information that obviously affected these two factors most. For example, the sensory status is binary, e.g. presence or loss of protective sensation, which reflects a rather coarse representation of information. More detailed measurement approaches, e.g. the monofilament test, could provide more information for a predictive risk model. However, the PEDIS system was designed as a DFU classification system rather than a tool supporting risk models, which explains the categorical design.

With respect to validity, AUC values ranging from 0.7 to 0.8 are considered acceptable [43, 44]. Thus, our risk models may be useful as clinical decision support tools to screen and stratify patients according to their six-month amputation incidence risk. Among the uninformed models, the PEDIS classification had a higher predictive value for any-amputation compared to major-amputation, as indicated by the AUC values (0.793 vs 0.765).

Incorporating prior information and thus reducing the variance, we were able to increase the predictive value of the major-amputation model (AUC: 0.766 vs 0.790).

### Bayesian modelling and clinical impact

**Fig. 2:**
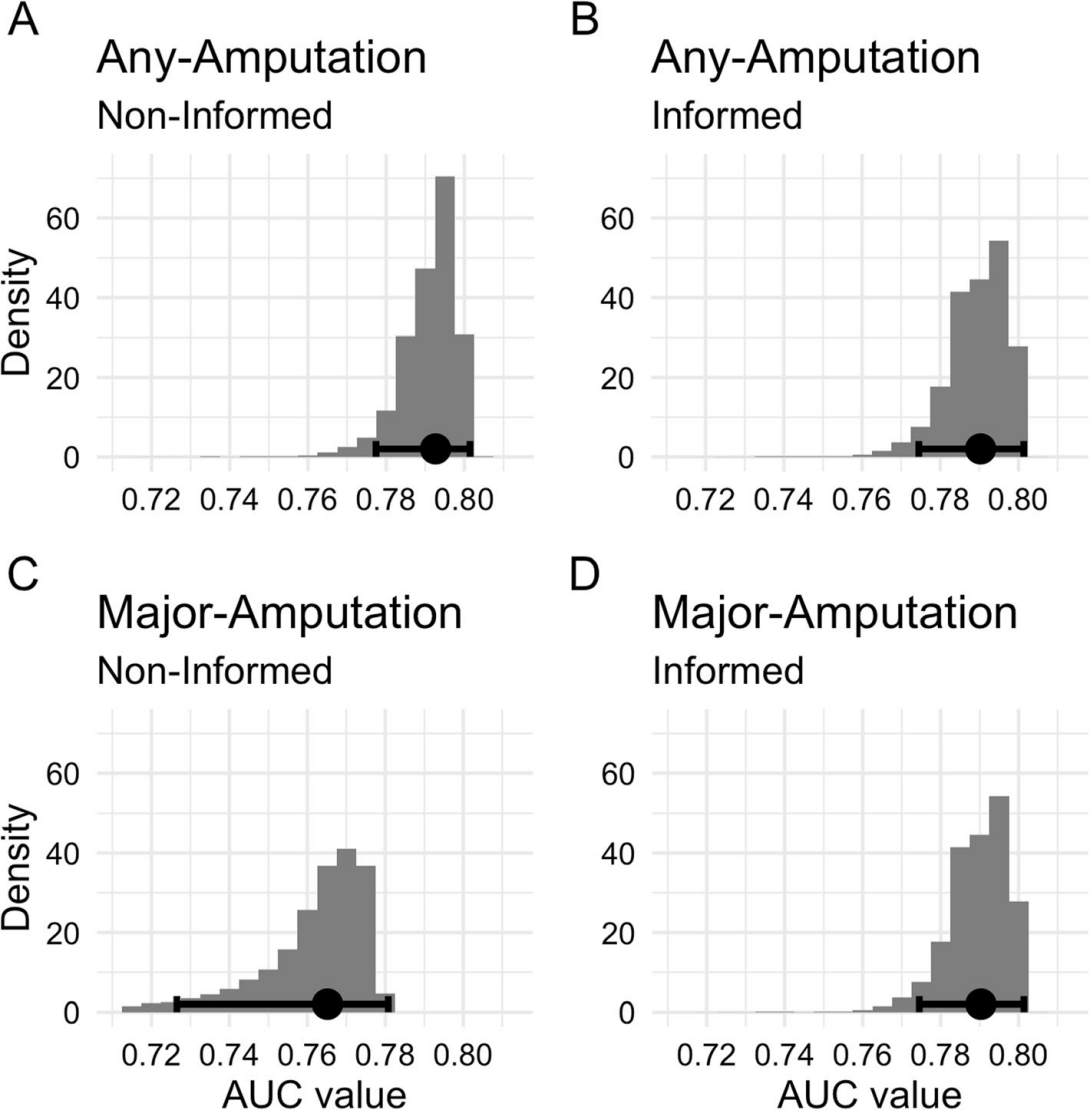
The posterior distributions of the AUC values. The error bars show the 95% highest density interval. The black square of each error bar shows the posterior AUC median estimate. Risk models: **a** uninformed any-amputation; **b** informed any-amputation; **c** uninformed major-amputation; **d** informed major-amputation

**Fig. 3:**
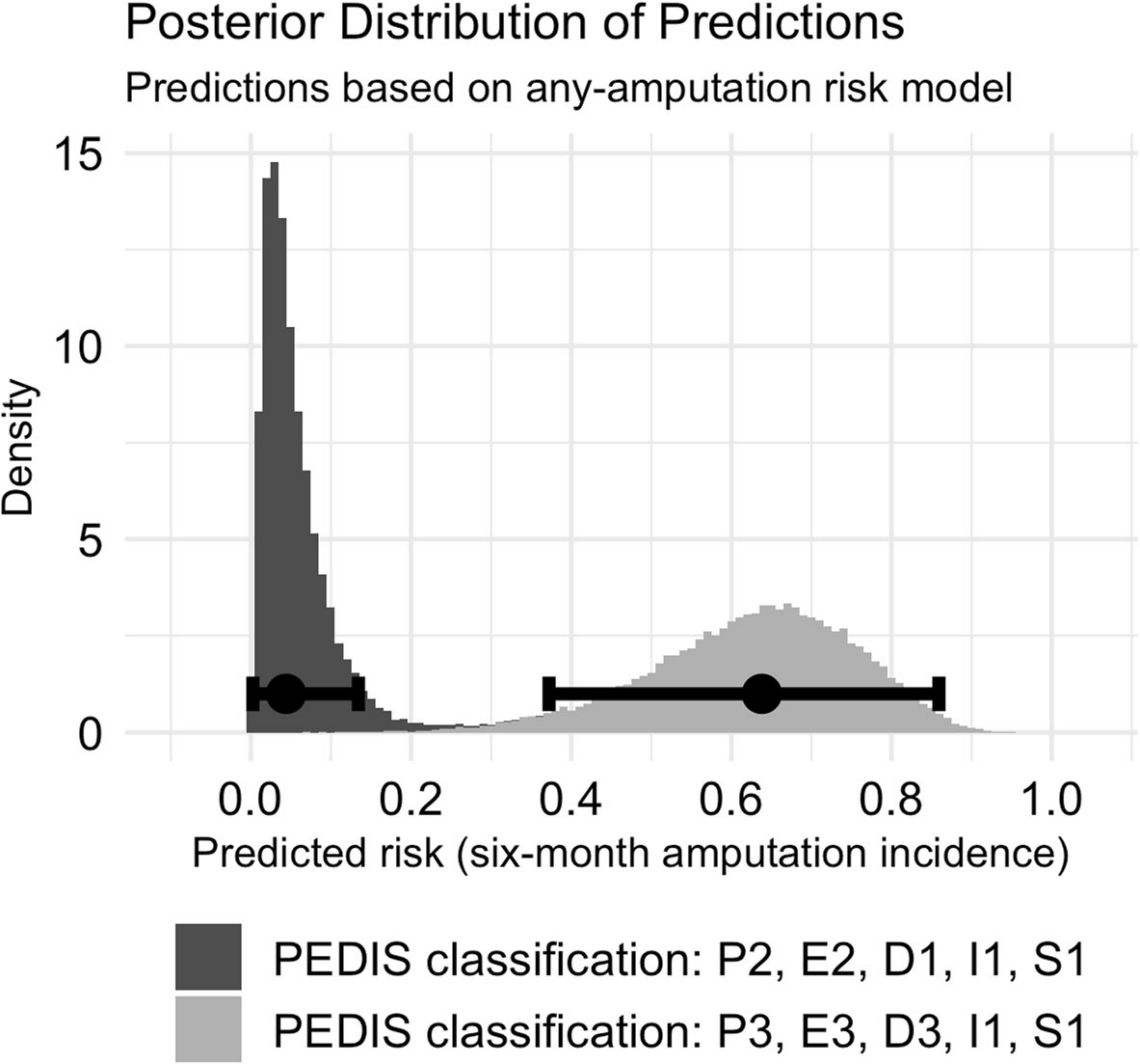
Example of the posterior distributions of the predicted amputation risk based on the informed any-amputation risk model. The solid dot and the error bar indicate and the corresponding posterior median risk estimate and the 95% HDI. The amputation risk for the lower PEDIS classification is 4.4% [95% HDI 0.2–13%] and for the higher PEDIS classification 63% [95% HDI 37.2–85.9%]

In this study, we chose to analyse the data using Bayesian modelling to make use of three key features that are not available for classical statistical techniques:

First, as described in the previous section, we took advantage of available prior knowledge about DFU risk factors. Second, a further advantage of the Bayesian framework is that it can be seen in a diachronic light. In this context, a Bayesian model evolves through time: As new data becomes available, the posterior distributions serve as informed priors to a consecutive Bayesian analysis. This approach opens the door of pooling model information across institutions without sharing sensitive patient information but only the posterior coefficient distributions. To promote this feature, we published the posterior distributions of the presented models. Third, opposed to classical models which commonly provide a single point estimate and a confidence interval, Bayesian models provide richer information, such as the distribution of the model coefficients (Fig. 1) and the AUC value (Fig. 2). Moreover, the distribution of the predicted values is available. Presenting the predictions in this way (Fig. 3), trained clinicians obtain more information from the distribution, such as the most probable estimated risk and the uncertainty associated with this estimate. Moreover, the interpretation of the posterior distribution could be more intuitive compared to classical estimates, such as confidence intervals [45]. In this context, the Bayesian framework may provide a sound basis for future transparent clinical decision support. As they require calculations based on the MCMC samples, such decision support cannot be provided manually but should rather be embedded in digital software products used in daily clinical routine. Furthermore, clinicians need to be trained to make appropriate use of uncertainty and the interpretation of intervals.

### Limitations

The findings should not be interpreted without knowing the specific limitations of this study. The study is based on a comparably small sample size, which we collected in a single wound care centre. This raises concerns about their external validity. This deficiency can be mitigated via the Bayesian approach of integrating the existing available scientific knowledge via prior distributions, which was exercised successfully in this study. To account for limited comparability, we designed our priors to be conservative estimates of a priori knowledge. More studies of this kind with more specific and detailed prior information than incorporated into this study would further enhance the generalisability of these findings while preserving the local findings. These local findings might have their own right and usefulness within the context of a local learning health system where local populations are of interest.

We aimed to examine the prognostic value of the PEDIS system because it is a widely applied and accepted classification in clinical DFU care. Although the AUC values of the PEDIS risk models are considered acceptable, they should be used carefully in clinical decision processes because it is important to remember that the primary use case for the PEDIS system is DFU classification rather than prediction. Thus, to further improve the prognostic value of a risk model, additional DFU characteristics may be helpful, e.g. ulcer history and diabetes onset, which we did not include. Furthermore, future risk models should also consider psychosocial variables as they are closely linked to DFU healing [46–48].

## Conclusion

In summary, we developed PEDIS risk models predicting the six-month amputation risk for any- and major-amputation using a Bayesian framework. Among the PEDIS risk factors, perfusion status, ulcer extent and ulcer depth were most closely related to both amputation outcomes. Both amputation risk models showed acceptable prognostic accuracy but needed additional information to be incorporated for improving the clinical prognostic values. Furthermore, we encourage future research initiatives with related scientific goals to make use of our Bayesian posterior distributions and embody them in their own Bayesian analysis.

## Supplementary information

**Additional file 1:.** The effective sample size for each predictor and model. Effective sample sizes > 10,000 are considered as sufficient.

**Additional file 2:.** The table shows the coefficients and 95% HDI of univariate models, that is, for each predictor, a distinct Bayesian logistic regression model was created. Coefficients are the median of the posterior distribution.

**Additional file 3:.** The online appendix is available at: https://jnshsrs.github.io/diabetic-foot/appendix.html.

**Additional file 4:.** The script with the complete analysis is available at: https://jnshsrs.github.io/diabetic-foot/analyis-bayes-models.html.

## Data Availability

The datasets generated during the current study are available in the GitHub repository at https://github.com/jnshsrs/diabetic-foot/tree/master/models.

## Abbreviations

DM: Diabetes Mellitus
LEA: Lower Extremity Amputation
DFU: Diabetic Foot Ulcer
PEDIS: Perfusion, Extent, Depth, Infection, Sensation
AUC: Area under the (Receiver Operating Characteristic) Curve
