# Supplementary material for: Predicting the amputation risk for patients with diabetic foot ulceration – a Bayesian decision support tool
Source: BMC Med Inform Decis Mak. 2020 Aug 24;20:200. doi: 10.1186/s12911-020-01195-x (PMC7446175; doi:10.1186/s12911-020-01195-x)
Supplement: Supplementary file 1 — Additional file 1:. The effective sample size for each predictor and model. Effective sample sizes > 10,000 are considered as sufficient. [file 12911_2020_1195_MOESM1_ESM.docx]

| **Predictor** | **Any-Amputation** | |  | **Major-Amputation** | |
| --- | --- | --- | --- | --- | --- |
|  | Non-Informed | Informed |  | Non-Informed | Informed |
| Intercept | 26,516 | 25,945 |  | 21,569 | 18,853 |
| Perfusion | 24,728 | 29,933 |  | 26,686 | 28,542 |
| Extent | 25,087 | 23,116 |  | 22,283 | 17,698 |
| Depth | 26,378 | 27,226 |  | 23,718 | 24,427 |
| Infection | 28,326 | 26,024 |  | 28,822 | 28,594 |
| Sensation | 32,397 | 33,476 |  | 30,906 | 31,812 |
| Age | 28,275 | 28,278 |  | 29,617 | 29,640 |
| Gender | 31,458 | 30,840 |  | 30,830 | 31,760 |

Appendix 1 The effective sample size for each predictor and model. Effective sample sizes > 10,000 are considered as sufficient.
