# Supplementary material for: Predicting the amputation risk for patients with diabetic foot ulceration – a Bayesian decision support tool
Source: BMC Med Inform Decis Mak. 2020 Aug 24;20:200. doi: 10.1186/s12911-020-01195-x (PMC7446175; doi:10.1186/s12911-020-01195-x)
Supplement: Supplementary file 2 — Additional file 2:. The table shows the coefficients and 95% HDI of univariate models, that is, for each predictor, a distinct Bayesian logistic regression model was created. Coefficients are the median of the posterior distribution. [file 12911_2020_1195_MOESM2_ESM.docx]

| **Predictors** | **Any Amputation** | | |  | **Major Amputation** | | |
| --- | --- | --- | --- | --- | --- | --- | --- |
|  | Non-Informed |  | Informed |  | Non-Informed |  | Informed |
|  | Beta-Coefficient (95% HDI) |  | Odds Ratio (95% HDI) |  | Beta-Coefficient (95% HDI) |  | Odds Ratio (95% HDI) |
| Perfusion | 1.100  [0.717 to 1.501] |  | 3.004  [2.049 to 4.488] |  | 0.829  [0.334 to 1.318] |  | 2.291  [1.397 to 3.737] |
| Extent | 1.948  [1.192 to 2.851] |  | 7.015  [3.293 to 17.309] |  | 1.753  [0.518 to 3.194] |  | 5.769  [1.679 to 24.374] |
| Depth | 1.174  [0.549 to 1.785] |  | 3.234  [1.732 to 5.963] |  | 1.544  [0.464 to 2.780] |  | 4.682  [1.590 to 16.122] |
| Infection | 0.092  [-0.197 to 0.402] |  | 1.097  [0.821 to 1.495] |  | 0.284  [-0.105 to 0.746] |  | 1.329  [0.901 to 2.109] |
| Sensation | 0.191  [-0.565 to 1.099] |  | 1.211  [0.568 to 3.001] |  | 0.270  [-0.729 to 1.919] |  | 1.310  [0.482 to 6.812] |
| Age | 0.027  [0.003 to 0.048] |  | 1.027  [1.003 to 1.049] |  | 0.017  [-0.013 to 0.049] |  | 1.017  [0.987 to 1.050] |
| Gender | 0.209  [-0.391 to 0.863] |  | 1.232  [0.676 to 2.370] |  | 0.119  [-0.621 to 0.988] |  | 1.126  [0.537 to 2.685] |

Appendix 2 The table shows the coefficients and 95% HDI of univariate models, that is, for each predictor, a distinct Bayesian logistic regression model was created. Coefficients are the median of the posterior distribution.
