# Supplementary material for: Predicting the amputation risk for patients with diabetic foot ulceration – a Bayesian decision support tool
Source: BMC Med Inform Decis Mak. 2020 Aug 24;20:200. doi: 10.1186/s12911-020-01195-x (PMC7446175; doi:10.1186/s12911-020-01195-x)
Supplement: Supplementary file 4 — Additional file 4:. The script with the complete analysis is available at: https://jnshsrs.github.io/diabetic-foot/analyis-bayes-models.html. [file 12911_2020_1195_MOESM4_ESM.docx]

Appendix 4: The script with the complete analysis is available at: <https://jnshsrs.github.io/diabetic-foot/analyis-bayes-models.html>
